# Transfer of Maternal Antimicrobial Immunity to HIV-Exposed Uninfected Newborns

**DOI:** 10.3389/fimmu.2016.00338

**Published:** 2016-08-31

**Authors:** Bahaa Abu-Raya, Kinga K. Smolen, Fabienne Willems, Tobias R. Kollmann, Arnaud Marchant

**Affiliations:** ^1^Department of Pediatrics, Division of Infectious Diseases, University of British Columbia, Vancouver, BC, Canada; ^2^Institute for Medical Immunology, Université Libre de Bruxelles, Charleroi, Belgium

**Keywords:** HIV infection, pregnancy, HIV-exposed uninfected, newborn, IgG, placenta, inflammation

## Abstract

The transfer of maternal immune factors to the newborn is critical for protection from infectious disease in early life. Maternally acquired passive immunity provides protection until the infant is beyond early life’s increased susceptibility to severe infections or until active immunity is achieved following infant’s primary immunization. However, as reviewed here, human immunodeficiency virus (HIV) infection alters the transfer of immune factors from HIV-infected mothers to the HIV-exposed newborns and young infants. This may relate to the immune activation in HIV-infected pregnant women, associated with the production of inflammatory cytokines at the maternofetal interface associated with inflammatory responses in the newborn. We also summarize mother-targeting interventions to improve the health of infants born to HIV-infected women, such as immunization during pregnancy and reduction of maternal inflammation. Maternal immunization offers the potential to compensate for the decreased transplacentally transferred maternal antibodies observed in HIV-exposed infants. Current data suggest reduced immunogenicity of vaccines in HIV-infected pregnant women, possibly reducing the protective impact of maternal immunization for HIV-exposed infants. Fortunately, levels of antibodies appear preserved in the breast milk of HIV-infected women, which supports the recommendation to breast-feed during antiretroviral treatment to protect HIV-exposed infants.

## Introduction

Young infants are vulnerable to severe morbidity and mortality caused by infectious diseases. Every year, more than 1 million children worldwide die from infections during the first 28 days of life (1, 2). This susceptibility to infectious pathogens involves multiple factors, including lack of immunological memory in the newborn infant and the regulatory responses required to adapt the immune system to the transition from a relatively sterile to a microbe-rich environment (3, 4). The transfer of maternal immune factors, through the placenta and breast milk, to the fetus and young infant plays a critical role in augmenting defense against infectious pathogens and participates in the establishment of immune homeostasis in early life. Specifically, maternal antibodies are actively transferred to the fetus *in utero* and provide protection against pathogens that are prevalent in the community (5), and breast-feeding extends the time for transfer of maternal immune factors, providing important protection against infectious disease morbidity and mortality in infancy (6, 7).

Chronic maternal infections can alter the immune factors that are transferred to the young infant, and thereby modulate their susceptibility to homologous or heterologous infectious pathogens (8). Human immunodeficiency virus (HIV) infection is known to have a profound impact on B lymphocyte and antibody responses to pathogens and vaccines (9, 10). These alterations are linked to immune activation and are improved by antiretroviral (ARV) therapy (10–12). Studies suggested that both HIV infection and pregnancy promote the activation of the immune system (13), and HIV infection alters the transfer of maternal immune factors to the newborn and young infant. As reviewed elsewhere in this research topic, clinical and epidemiological studies have shown that infants born to HIV-infected women, but not infected by HIV, are at increased risk of severe infections, particularly during the first year of life (14). Although the mechanisms underlying this increased susceptibility have not yet been identified, alterations in the transfer of maternal immune factors could play a central role. As severe infections observed in HIV-exposed uninfected (HEU) infants involve multiple pathogens, including bacteria, viruses, and parasites, the immune factors involved should have the potential to impact defenses against a broad spectrum of microbes (15–18).

The aim of this article is to review the current knowledge on the transfer of immune factors from HIV-infected mothers to HEU infants through the placenta and breast milk and to discuss maternal interventions that could improve the health of these children. The transfer of HIV-specific immunity is not discussed in this review. As the definition of HEU requires follow-up of HIV-exposed infants to confirm the absence of transmission, this term will only be used when HIV-exposed infants were confirmed uninfected. Whenever these data are not available from the referred studies the term “HIV-exposed infant” will be used.

## Impact of Maternal HIV Infection on the Transplacental Transfer of Antibodies

Immunoglobulin G (IgGs) are specifically transported from maternal to fetal blood *via* the neonatal Fc receptor (FcRn) expressed in placental syncytiotrophoblasts (19). Most of this transfer occurs during the third trimester of pregnancy (19, 20). The efficiency of IgG transfer (measured as the ratios between cord blood and maternal blood antibody levels) differs between antibodies targeting different antigens or pathogens and ranges from up to 200% for pertussis and 70% for Group B *Streptococcus* (GBS) (21–23). Although direct evidence for this is limited, this antigen-specific variability is, at least partly, related to differences in the efficiency of the transfer of IgG subclasses. The highest transfer is observed for IgG1 that is predominantly induced by protein antigens (e.g., pertussis), whereas the lowest transfer is observed for IgG2 that is predominantly induced by polysaccharide antigens (e.g., GBS capsular antigen) (24–26). In early 1990s, studies of Brazilian women indicated that although HIV-infected women had higher total IgG levels than HIV-uninfected mothers at delivery, the transplacental transfer of total as well as antigen-specific IgG to HIV-exposed newborns was reduced (27, 28). These early studies were confirmed by many other investigators and extended to a number of pathogen and vaccine antigens (Table 1). To date, the mechanism underlying this reduced transfer remains poorly understood. The inverse association observed between maternal hypergammaglobulinemia and IgG transfer ratios has been interpreted as evidence that maternal IgG may saturate the placental FcRn (28–30). As our understanding of the mechanisms underlying transplacental transfer of IgG remains very poor, this model remains speculative.

**Table 1:** **Transplacental transfer of pathogen- and vaccine-specific antibodies from HIV-infected pregnant women to the newborn**.

Settings	Main results
**Transfer of antibacterial antibodies**	
***Streptococcus pneumonia* (Spn)**	
Brazil, 1989–1992	Transplacental transfer of total anti-PCP IgG was reduced in newborns of HIV-infected (*n* = 46) as compared with HIV-uninfected mothers (*n* = 53)^a^ (27)
Malawi, 1993–1994	Maternal HIV infection was not associated with reduced placental transfer or lower cord total anti-PCP IgG^a^ (35)
South Africa, 2009–2010	At birth, HEU newborns (*n* = 46) had significantly lower total anti-PCP IgG levels than HU newborns (*n* = 54) (31)
United Kingdom, 2011–2012	Transplacental transfer of total anti-PCP IgG was reduced by 28% in HEU (*n* = 12) as compared with HU newborns (*n* = 96). HEU infants (*n* = 13) had lower levels of total anti-PCP IgG compared with HU (34)
India, 2002–2007; Bangladesh, 2004–2005	HIV-exposed infants (*n* = 74, India) compared with HU (*n* = 98, Bangladesh) had lower umbilical cord GMTs for serotype-specific Spn antibodies^b^ and lower geometric mean ratio of cord: maternal antibody concentrations for all serotypes^b^ except serotype 6B. The proportion of umbilical cord samples with Spn IgG ≥ 0.35 μg/ml, was lower in HIV-exposed infants than HU for all serotypes^b^ (33)
***Haemophilus influenzae* type b (Hib)**	
South Africa, 2009–2010	At birth, HEU infants (*n* = 46) had significantly lower levels of anti-Hib IgG than HU infants (*n* = 54). There was lower proportion of HEU infants with levels considered to be protective against Hib (anti-Hib IgG titers > 1 mg/l) as compared with HU infants (31)
Uganda, 2010–2013	Transplacental transfer of anti-Hib IgG in HIV-infected mothers was 43%. Ninety percent of the infants had anti-Hib IgG levels above the threshold for long-term protection (>1.0 μg/ml) (38)
United Kingdom, 2011–2012	The transplacental transfer of anti-Hib IgG was reduced by 61%% in HEU (*n* = 12) as compared with HU infants (*n* = 96) (34)
**Group B *Streptococcus* (GBS)**	
South Africa, 2009–2011	HIV infection was associated with a reduction in placental transfer of anti-GBS antibodies to serotypes II and V. HEU infants (*n* = 45) had lower antibody GMTs to GBS capsular serotypes (Ia, Ib, II, III, and V) than HU infants (*n* = 54). Antibody GMTs to all serotypes remained lower at 16 weeks of age in HEU as compared with HU infants (39)
South Africa, 2013	Antibodies against GBS capsular serotypes (Ia, Ib, III, and V) and the cord-maternal ratio (serotypes Ia and III) were lower in HEU (*n* = 83) than in HU (*n* = 81) newborns. The cord blood median surface-protein antibody concentrations were lower in HEU compared with HU newborns for selected GBS surface proteins (40)
**Tetanus (TT)**	
Brazil, 1989–1992	Transplacental transfer of anti-TT IgG was reduced in sera of newborns of HIV-infected (*n* = 46) as compared with -uninfected mothers (*n* = 53)^a^ (28)
Malawi, 1993–1994	Maternal HIV infection was not associated with lower cord anti-TT IgG or reduced placental transfer of anti-TT IgG^a^ (35)
Kenya, 1996–1997	There was a 52% reduction in anti-TT IgG concentrations in newborns born to HIV-infected (*n* = 87) as compared with -uninfected (*n* = 617) women. Increased risk for seronegativity (anti-TT IgG < 0.1 IU/ml) in newborns of women infected with HIV compared with -uninfected women (29)
South Africa, 2009–2010	Tranplacental transfer of anti-TT IgG was reduced in HIV-infected (*n* = 46) as compared with -uninfected women (*n* = 54). HEU infants had lower anti-TT IgG at birth as compared with HU infants. There was significant lower proportion of HEU infants with sero-protective levels against tetanus (anti-TT IgG > 0.1 IU/ml) (31)
United Kingdom, 2011–2012	Anti-TT IgG levels were similar between HEU and HU infants at birth (34)
Nigeria, date not provided	HIV-exposed newborns (*n* = 10) were more likely to have cord:maternal transfer ratio of anti-TT IgG < 1 than HU newborns (*n* = 152). HIV-exposed newborns were more likely to be seronegative (anti-TT IgG < 0.1 IU/ml) than HU newborns (43)
**Pertussis**	
South Africa, 2009–2010	Transplacental transfer of pertussis antibodies was reduced in HIV-infected women (*n* = 46) as compared with -uninfected women (*n* = 54). HEU newborns had lower levels of pertussis-specific antibodies compared with HU infants (31)
United Kingdom, 2011–2012	Transplacental transfer of collective IgG response to pertussis antigens (PT and FHA) was reduced by 32% in HEU (*n* = 12) as compared with HU newborns (*n* = 96). Pertussis-specific antibody levels were similar in HEU and HU newborns (34)
**Transfer of antiviral antibodies**	
**Measles**	
Brazil, 1989–1990	No difference in anti-measles IgG titers between newborns of HIV-infected (*n* = 16) and -uninfected (*n* = 18) pregnant women. The number of newborns with protective anti-measles antibodies was lower among newborns of HIV-infected of -uninfected mothers^a^ (27)
Brazil, 1989–1992	Transplacental transfer of anti-measles IgG was reduced in sera of newborns of HIV-infected (*n* = 46) as compared with -uninfected mothers (*n* = 53)^a^ (28)
Malawi, 1993–1994	Maternal HIV infection was not associated with lower cord anti-measles antibodies or reduced transplacental transfer of anti-measles antibodies^a^ (35)
Kenya, 1996–1997	Maternal HIV infection was associated with 15.5% reduction in the transfer of anti-measles antibodies to HEU (*n* = 91) as compared with HU newborns. HEU newborns had a 35% reduction in measles antibody levels and a fourfold increased risk of being seronegative than HU newborns (46)
**Polio**	
Brazil, 1989–1990	Anti-poliovirus 1 and 2 titers in umbilical cord sera were lower in newborns of HIV-infected (*n* = 16) than -uninfected women (*n* = 18)
	A reduction in transplacental transfer of type 2 poliovirus antibodies was demonstrated in newborns of women infected with HIV than -uninfected women^a^ (27)
**Varicella zoster virus (VZV)**	
Brazil, 1989–1992	Transplacental transfer of anti-VZV IgG was reduced in newborns of HIV-infected (*n* = 46) as compared with -uninfected women^a^ (*n* = 53) (28)
**Transfer of antimalarial antibodies**	
Kenya, 1997	Maternal HIV infection was associated with reduced transfer and reduced newborn levels of antibodies to the (NANP) 5 antigen but not of other malaria antigens^a^ (42)
Kenya, 1996–2000	Reduced levels of antibodies against CSP, liver-stage antigen 1, and RAP1 at birth in newborns of HIV-infected women (*n* = 254) (43)
Mozambique, 2003–2006	Maternal HIV infection was associated with decreased levels of cord IgG1 and IgG3 against MSP1 and lysate, with cord IgG1 against apical membrane antigen 1 (AMA1), and with cord IgG3 against EBA175. Maternal HIV infection was associated with reduced transfer of IgG1 against AMA1, of IgG1 and IgG3 against lysate, and of IgG3 against MSP1 and EBA175 (48)

*HIV, human immunodeficiency virus; PCP, pneumococcal capsular polysaccharide; IgG, immunoglobulin G; HEU, HIV-exposed uninfected; HU, HIV-unexposed; GMT, geometric mean titers; Hib, Hemophilus influenzae B; GBS, group B Streptococcus; CSP, circumsporozoite protein; RAP, rhoptry associated protein; MSP, merozoite surface protein; AMA, apical membrane antigen; EBA, erythrocyte-binding antigen*.

*^a^Infants were not followed with HIV testing*.

*^b^Serotypes 1, 4, 5, 6B, 9V, 14, 18C, 19F, and 23F*.

### Transfer of Antibacterial Antibodies

#### *Streptococcus* *pneumoniae*

Studies consistently showed that maternal HIV infection is associated with a decrease in the transfer of antibodies to *Streptococcus pneumoniae*. In South Africa, HIV-infected mothers had lower titers of total pneumococcal capsular polysaccharide (PCP) antibodies and a 15% reduction in antibody transfer ratios as compared with HIV-negative mothers; this resulted in lower PCP antibody titers in their newborns (31). In Malawi, the transfer of PCP antibodies was inversely correlated with HIV viral load and correlated with CD4 counts in ARV therapy-naive pregnant women (32). In South Asia, HIV-infected mothers had lower titers of antibodies to multiple PCP serotypes (1, 4, 5, 9V, 14, 18C, 19F, and 23F) and a 23–48% reduction in antibody transfer ratios as compared with HIV-negative mothers (33). In United Kingdom (UK), HIV-infected mothers had similar levels of anti-PCP antibodies compared with HIV-negative mothers but had a 28% reduction in antibody transfer ratios; this again resulted in lower antibody titers in their newborns (34). In these studies, most pregnant women had not received pneumococcal vaccines, i.e., measured antibodies were induced by natural exposure to *S. pneumoniae*. However, while the transfer of naturally occurring (i.e., infection-induced) PCP IgG was reduced by about 20% in HIV-infected mothers (28, 35), antibody transfer ratios following immunization of pregnant women in Brazil with a pneumococcal polysaccharide vaccine was also reduced to only 46–72% as compared with unexposed newborns (36). Thus, infection-induced as well as vaccine-induced anti-PCP IgG transfer to her newborn is reduced in HIV-infected women. The clinical relevance of reduced PCP antibody transfer to HIV-exposed infants has not been clearly established. In Malawi, respiratory tract infections were more common among children with lower PCP antibodies, but no difference in mortality was observed (32, 37). A study in Belgium showed that HEU children are at high risk of invasive pneumococcal infections, but the association with the level of maternal antibodies was not assessed (16).

#### *Haemophilus influenzae* Type b

Maternal HIV infection is also associated with a reduced transplacental transfer of *Haemophilus influenzae* type b (Hib) antibodies. In South Africa, transfer of Hib antibodies to HEU newborns was reduced by 23% as compared with HIV-unexposed newborns, and this was associated with 35% reduction in the proportion of newborns with protective antibody levels (anti-Hib IgG > 1 mg/l) (31). In Uganda, 43% of maternal Hib antibodies were transplacentally transferred to HEU newborns; however, 90% of HEU newborns had protective antibody levels (38). Interestingly, these antibodies were mostly of IgG1 subclass, an unexpected finding given the polysaccharide nature of the Hib antigen. In UK, maternal HIV infection was associated with a 61% reduction in the transfer of Hib antibodies, but antibody levels were not significantly different in HEU and HIV-unexposed newborns (34). In these studies, antibodies were induced by natural infection. No data are available regarding the transfer of Hib vaccine-induced antibodies. Also, the clinical impact of the decreased antibody transfer has not been established.

#### Group B *Streptococcus*

Data indicating that HEU infants are at high risk of invasive GBS infections stimulated studies that focused on the impact of maternal HIV in the transfer of anti-GBS immunity (15). In South Africa, maternal HIV infection was associated with lower levels of antibodies to GBS capsular polysaccharide antigens (serotypes Ia, Ib, II, III, and V) and a 58 and 32% reduction in transfer ratios of antibodies to serotypes II and V, respectively (39). These results were confirmed in another study from South Africa, which also demonstrated reduced levels of antibodies to GBS capsular polysaccharide antigens and surface proteins in HIV-infected mothers and their newborns (40). However, the transfer ratios of antibodies to GBS proteins were not different in HIV-infected and -uninfected mothers. No association was observed between maternal antibody levels or transfer ratios and CD4 counts or viral load (40). A more recent study also conducted in South Africa demonstrated that maternal HIV infection reduces antibody response to a GBS glycoconjugate vaccine, but does not impact the transfer ratios of vaccine-induced antibodies to the HIV-exposed newborns (41).

#### Tetanus

The impact of maternal HIV infection on the transplacental transfer of tetanus toxoid (TT) antibodies varies between studies and populations. Early studies in Brazil indicated reduced transfer of TT antibodies in HIV-infected mothers, but this was not confirmed in a study in Malawi (28, 35). In Kenya, TT antibody titers were 38% lower in HIV-infected than in -uninfected women at delivery and 52% lower in HIV-exposed as compared with HIV-unexposed newborns (29). Similar results were obtained in a second, but not in a third, study in Kenya (42, 43). The reasons for these discrepant results are not clear but could involve interactions with other pathogens coinfecting pregnant women and affecting transplacental transfer of maternal antibodies, including malaria (43). In South Africa, a 27% reduction of TT antibody transfer ratios was observed in HIV-infected as compared with -uninfected women, and this resulted in a 30% reduction in seroprotection rates in HEU as compared with HIV-unexposed newborns (31). In UK, maternal HIV infection was associated with a 32% reduction in transfer of TT antibodies, but antibody levels were not significantly different in HEU and HIV-unexposed newborns (34). Recent data from Nigeria showed that HIV-exposed newborns were nearly 34 times more likely than HIV-unexposed newborns to have seronegative TT titers in cord blood (anti-TT IgG < 0.1 IU/ml) (44). Although this has not been determined, these data suggest that HEU newborns may be at higher risk of tetanus than children born to HIV-negative women (45).

#### Pertussis

Maternal HIV infection is associated with reduced transplacental transfer of pertussis antibodies, but the resulting antibody levels in HEU newborns differ between populations. In South Africa, HIV-infected mothers had a 40% reduction in pertussis antibody transfer ratios, and this resulted in lower levels of pertussis antibodies in HEU as compared with unexposed newborns (31). A marked reduction in the transplacental transfer of pertussis antibodies was also observed in UK, but no significant difference in antibody levels was observed between HEU and HIV-unexposed newborns (34). The clinical implications of these observations have not been established.

### Transfer of Antiviral Antibodies

#### Measles Virus

In Brazil, HEU newborns had 20% lower measles antibody levels than HIV-unexposed newborns (28). In Kenya, maternal HIV infection was associated with a 16% reduction in the transfer ratios of measles antibodies; HEU newborns had a 35% reduction in measles antibody levels and a fourfold increase in the rate of measles seronegativity (46). In contrast, HIV-infected pregnant women in Malawi had higher measles antibody levels than -uninfected mothers and their newborns had similar antibody levels than unexposed newborns (35). In another study, in Kenya, high maternal HIV viral load was correlated with decreased transfer ratios of measles antibodies (47).

#### Polio and Varicella Zoster Viruses

Limited data are available regarding the transfer of polio or varicella zoster virus (VZV) antibodies in HIV-infected mothers. In Brazil, HEU newborns had lower levels of polio antibodies than HIV-unexposed newborns and decreased transfer ratios were demonstrated for polio virus 2 (27). Higher levels of VZV antibodies were detected in HIV-infected pregnant women as compared with -uninfected controls (28). But, while transplacental transfer of VZV antibodies was reduced by maternal HIV infection, similar antibody levels were detected in HEU and unexposed newborns (28).

### Transfer of Antimalarial Antibodies

Limited data are available regarding the transplacental transfer of maternal antibodies directed against parasite antigens. A study in Kenya analyzed the transfer of antibodies to a series of *Plasmodium falciparum* antigens. Transfer ratios were about 100% for all tested antigens, and maternal HIV infection was associated with a reduction in transfer of only one antigen, the circumsporozoite protein (NANP) 5 (42). In another study, in Kenya, HIV-exposed newborns had lower levels of antibodies to several pre-erythrocytic and erythrocytic-stage antigens as compared with HIV-unexposed newborns (43). In Mozambique, maternal HIV infection was associated with reduced transfer rates of IgG1 and IgG3 to parasite lysate and erythrocytic-stage antigens (48).

### Impact of Maternal HIV Infection on the Quality of Transferred Antibodies

Whether maternal HIV infection alters the quality of antibodies transferred to the newborn remains largely unexplored. In South Africa, reduced antibody-mediated complement C3b/iC3b deposition on GBS serotypes was detected in HIV-infected as compared with HIV-uninfected mothers and HEU as compared with HIV-unexposed newborns (39). However, the intrinsic complement deposition activity of the transferred antibodies was not assessed in this study. In a small study, Farouk et al. observed reduced measles neutralizing activity of antibodies from HIV-exposed as compared with unexposed newborns, suggesting the intriguing possibility that maternal HIV infection alters the quality of antibodies transferred to their newborns (49).

## Transfer of Inflammatory Mediators from HIV-Infected Pregnant Women to the Fetus

The production of inflammatory mediators induced by HIV infection may alter the immune homeostasis of healthy pregnancy, and this may have important consequences on the outcome of the pregnancy itself and on the health of HEU children. Maternal HIV infection is associated with an increased risk of preterm birth, a complication likely related to inflammation at the maternofetal interface (50, 51). Inflammation in the fetus may result in fetal inflammatory response syndrome, but the incidence of this syndrome in HIV-exposed fetuses has not been reported (52, 53). Fetal inflammation may also alter the neurological development of HEU children and may participate in their increased susceptibility to severe infections (54–57).

### Inflammatory Responses in HIV-Infected Pregnant Women

Studies performed in the 1990s indicated increased serum levels of several inflammatory mediators, including tumor necrosis factor (TNF)-α, neopterin, and β2 microglobulin, in HIV-infected as compared with -uninfected pregnant women, and these levels increased from the first to the third trimester of pregnancy (58). These observations were confirmed by later studies indicating that HIV-infected pregnant women have increased levels of inflammatory mediators in the plasma and in cervicovaginal fluid as compared with -uninfected pregnant women and to HIV-infected non-pregnant women, indicating that both HIV infection and pregnancy contribute to the production of inflammatory mediators (59). Studies suggest that peripheral blood cells may contribute to the production of inflammatory mediators in HIV-infected pregnant women (60). Whether these mediators could cross the placenta and reach the fetal circulation has not been established. *Ex vivo* placental perfusion models suggested that some cytokines could be transferred, but these observations have not been reproduced in other studies (61, 62). No study has examined the transfer of cytokines through the placenta of HIV-infected women. A recent study suggested that maternal HIV infection is associated with increased serum levels of inflammatory markers related to microbial translocation [lipopolysaccharide (LPS) modulators soluble CD14 and LPS-binding protein] and that these markers were associated with preterm delivery (51); however, the cord blood levels of these pro-inflammatory mediators were not different among newborns of HIV-infected and -uninfected women, and the ratio of transfer across the placenta was not provided.

### Placental Inflammation

Several studies suggest that HIV infection promotes inflammatory responses at the level of the placenta. Placental immune cells from HIV-infected women secrete higher levels of inflammatory cytokines, including interleukin (IL)-1β and IL-6, as compared with HIV-uninfected women (63). In one study, maternal HIV infection was associated with an increased production of inflammatory cytokines, including IL-1β, IL-6, and TNF-α, by placental trophoblasts (epithelial cells of fetal origin) (64). In another study, HIV-infection was associated with an increased production of interferon (IFN)-β by trophoblasts but with similar production of IFN-α, IFN-γ, and chemokines as compared with -uninfected pregnancies (65). These results suggest that specific inflammatory pathways may be activated at the placental level during HIV infection.

### Inflammation in HIV-Exposed Newborns

A restricted number of studies suggested that HIV-exposed newborns have signs of systemic inflammation. In South Africa, HIV-exposed newborns had increased serum levels of neopterin, suggesting myeloid cell activation (66). Interestingly, neopterin levels where higher in newborns of mothers who received nevirapine prophylaxis. In Netherlands, increased levels of IL-1β and IL-8 were detected in the plasma of HIV-exposed as compared with unexposed newborns (67). Evidence for innate immune cell activation was provided by a study conducted in South Africa where peripheral blood innate immune cells of HEU infants mounted an enhanced inflammatory response to microbial products as compared with HIV-unexposed infants (68). In the USA, HEU newborns also displayed higher expression of T-cell costimulatory molecules by dendritic cells following LPS stimulation as compared with unexposed newborns (69). In contrast, IL-12 responses were lower in HEU newborns, suggesting a potential impact on the quality of T-cell responses (70). Altered cytokine responses to lectin stimulation were also observed in Brazil (60). So, although a clear picture of the pathways involved and of the underlying mechanisms is not yet available, evidence indicates that peripheral blood cells of HEU newborns are phenotypically and functionally altered.

## Transfer of Maternal Immune Factors by Breast-Feeding

Breast milk contains antibodies and leukocytes that can participate in pathogen-specific immunity after birth. If the presence of HIV-specific antibodies in breast milk has been well studied, the impact of maternal HIV infection on the concentration of antibodies to other pathogens or vaccine antigens has been less well characterized (71–74). In Botswana, HIV-infected women had higher concentrations of total IgG, IgM, and IgA in breast milk as compared with -uninfected women; the levels in breast milk paralleled their hypergammaglobulinemia (75). However, different antigen specificities appeared to be differently affected, with IgG to *H. influenzae* detected at higher concentrations and IgG to *Campylobacter jejuni, Helicobacter pylori*, or *S. pneumoniae* detected at similar concentrations in breast milk of HIV-infected as compared with -uninfected women (75). These higher or preserved IgG concentrations in breast milk may compensate for the decreased transfer of maternal IgG through the placenta.

Very little data exist on the effect of maternal HIV infection on the cell-mediated and innate immunity in the breast milk; discussion of this is beyond the scope of this review.

## Maternal Interventions to Promote Immunity in HEU Infants

Interventions in HIV-infected mothers have been identified that have the potential to promote immunity in HEU infants. They include maternal immunization and reduction of maternal inflammation by ARV therapy (Table 2). The discussion about breast-feeding during ARV therapy is beyond the scope of this review.

**Table 2:** **Mother-targeted interventions to optimize HIV-exposed infants’ health**.

1. **Immunization of HIV-infected pregnant women during pregnancy** – **Immunization of HIV-infected pregnant women with influenza vaccine:** immunization with two doses of pandemic H1N1 monovalent vaccine induced similar seroprotection rates in HIV-infected pregnant women and their newborns at delivery. Seroprotection in HIV-exposed infants waned rapidly (76). Immunization with trivalent inactivated influenza vaccine (A/California/7/2009, A/Victoria/210/2009, and B/Brisbane/60/2008) induced lower seroprotection rates in HIV-infected as compared with -uninfected pregnant women, but similar vaccine efficacy (78). Lower maternal antibody transfer ratios were observed in immunized HIV-infected as compared with -uninfected women for the California and not for the two other vaccine strains (79) – **Immunization of HIV-infected pregnant women with pneumococcal vaccine:** immunization of pregnant women with a pneumococcal polysaccharide vaccine during the third trimester of pregnancy was associated with 46–72% antibody transfer ratios (36). Serotype-specific antibody levels waned rapidly in the HIV-exposed infants over the first 6 months of life with the estimated time to antibody levels <0.35 μg/ml for different serotypes (1, 3, 5, 6B, and 9V) ranging between 0.6 and 2.7 months (36) – **Immunization of HIV-infected pregnant women with GBS vaccine:** immunization with non-adjuvanted GBS capsular polysaccharide during pregnancy induced lower antibody response in HIV-infected than in -uninfected women (41). Transfer ratios were similar but antibody levels were lower in HIV-exposed as compared with unexposed newborns (41)
2. **Reduction of maternal inflammation** – Placental tissues from HIV-infected pregnant women on combined ARV treatment expressed lower levels of mRNA for TNF-α and IL-8 as compared with -uninfected women (82).

*HIV, human immunodeficiency virus; GBS, group B Streptococcus; ARV, antiretroviral; TNF-α, tumor necrosis factor; IL, interleukin*.

### Immunization of HIV-Infected Pregnant Women

Immunization of HIV-infected pregnant women offers the potential to compensate for the reduced transfer of maternal antibodies to HEU newborns. Yet, maternal immune responses may be affected both quantitatively and qualitatively by HIV infection. As a result, the benefit of this strategy has to be evaluated for each individual vaccine and vaccination strategy. The relatively few studies conducted on this topic have examined the response to influenza, tetanus, *S. pneumoniae*, and GBS vaccines.

#### Influenza

Immunization of pregnant women may protect the infant against influenza by reducing mother’s risk of influenza disease and by augmenting the transplacental transfer of influenza specific antibodies to the infant. Current evidence indicates reduced immunogenicity of inactivated influenza vaccines in HIV-infected as compared with -uninfected pregnant women and relatively rapid waning of maternal antibodies in HIV-exposed infants.

During the 2009 influenza season in the US, HIV-infected pregnant women on highly active ARV therapy (HAART) prior to or at study entry were immunized with two doses of pandemic H1N1 monovalent vaccine between 13 and 34 weeks of gestation (76). Antibody responses were lower than in HIV-uninfected historical controls. Seroprotection [hemagglutinin inhibition (HAI) titers ≥ 40] rates were similar in women and newborns at delivery (67 and 65%, respectively), indicating efficient transfer of maternal antibodies. However, maternal antibodies declined relatively rapidly in HIV-exposed infants, with seroprotection rates reaching only 26% in HIV-exposed infants at 3 months of age. A follow-up publication indicated a positive correlation between CD4 counts and antibody response to the first vaccine dose and minimal response to the second dose of vaccine (77).

In South Africa, a randomized placebo controlled trial measured the immunogenicity and efficacy of a trivalent inactivated influenza vaccine (A/California/7/2009, A/Victoria/210/2009, and B/Brisbane/60/2008) in HIV-infected and -uninfected pregnant women (78). Seroprotection rates (HAI titers) were lower in HIV-infected (43, 36, and 40% to California, Victoria, and Brisbane strains) as compared with HIV-uninfected pregnant women (73, 65, and 92% to California, Victoria, and Brisbane strains). However, vaccine efficacy was similar in both groups (57 and 50% in HIV-infected and -uninfected women, respectively). Transfer ratios ranged between 0.7 and 1.0 in HIV-uninfected and between 0.7 and 1.4 in HIV-infected immunized women, suggesting no negative impact of maternal HIV infection on antibody transfer. The transfer ratios of antibodies were higher in the placebo as compared with the vaccinated group for the Victoria strain in HIV-uninfected women and for California and Victoria strains in HIV-infected women. At birth, seroprotection rates were 81, 60, and 82% in children born to immunized uninfected women and 61, 43, and 79% (California, Victoria, and Brisbane strains, respectively) in children born to immunized HIV-infected women. A follow-up publication included formal comparisons of maternal antibody transfer ratios and indicated lower ratios in immunized HIV-infected as compared with -uninfected women for the California and not for the two other vaccine strains (79). Intriguingly, maternal antibodies declined more rapidly in HIV-unexposed as compared with HIV-exposed children, with an estimated mean antibody half-life ranging from 43 to 45 and 56 to 65 days, respectively (79).

Study of a relatively small cohort showed a lower immunogenicity of a trivalent influenza vaccine in HIV-infected as compared with -uninfected pregnant women and a negative correlation with the frequency of regulatory T-cells in the HIV-infected group (80).

#### Tetanus

Although maternal immunization with TT vaccine is the cornerstone of the World Health Organization (WHO) neonatal tetanus elimination program (81), the immunogenicity of TT immunization in HIV-infected pregnant women remains largely unexplored. In Kenya, titers of TT antibodies at delivery were 38% lower in HIV-infected as compared with -uninfected women (29). However, the proportion of women immunized with TT during pregnancy was not reported.

#### *Streptococcus* *pneumoniae*

In Brazil, immunization of HIV-infected pregnant women with a pneumococcal polysaccharide vaccine during the third trimester of pregnancy was associated with 46–72% antibody transfer ratios (36). Moreover, serotype-specific antibody levels waned rapidly in HIV-exposed infants over the first 6 months of life with an estimated time to antibody levels <0.35 μg/ml for different serotypes (1, 3, 5, 6B, and 9V) ranging between 0.6 and 2.7 months (36). This rapid waning of passive immunity could place HIV-exposed infants at risk for invasive pneumococcal disease during highly vulnerable period of early life.

#### Group B *Streptococcus*

The first study on the immunogenicity of a GBS vaccine in HIV-infected and -uninfected pregnant women was recently conducted in Malawi and South Africa (41). Women were immunized with a non-adjuvanted GBS capsular polysaccharide (serotypes Ia, Ib, and III) between 24 and 35 weeks of gestation. Antibody responses were lower in HIV-infected than in -uninfected women. Transfer ratios were similar (0.49–0.72) in HIV-infected and -uninfected women. At birth, HIV-exposed children had lower antibody titers than -unexposed children, and antibody decay was similar in the two groups during the first 6 weeks of life.

### Reduction of Maternal Inflammation by ARV Therapy

The impact of ARV therapy on placental inflammation induced by maternal HIV infection remains unclear. Historical studies indicating an increased expression of inflammatory mediators in the placenta of HIV-infected as compared with -uninfected pregnant women included patients who were untreated or received AZT prophylaxis (63–65). More recent studies reported an increased secretion of leukemia inhibiting factor (LIF), TNF-α, and IL-8 by placental explants from HIV-infected women under mono-, bi-, or tri-ARV therapy as compared with -uninfected women (82). However, placental tissues from these HIV-infected pregnant women expressed lower levels of mRNA for TNF-α and IL-8 as compared with -uninfected women. It was suggested that this discrepancy might be related to the fact that cytokine secretion by placental explants had been measured *ex vivo* after 24 h of culture and had, therefore, not been influenced by ARV therapy supporting the hypothesis that ARV drugs may affect the level of cytokines’ mRNA expression (82). In an *in vitro* model of placental histocultures, it was shown that AZT decreases levels of TNF-α mRNA expression in placental microexplants (83).

In a study conducted in Brazil, HIV-infected pregnant women under HAART had higher plasma levels of IL-1β and TNF-α and lower levels of the anti-inflammatory cytokine IL-10 as compared with HIV-infected untreated and -uninfected pregnant women (84). Interestingly, the same cytokine profile was observed in HAART-exposed newborns. These increased levels of inflammatory cytokines were associated with an increased production of TNF-α and IFN-γ and a decreased production of IL-10 by T lymphocytes *in vitro*.

Together, these data suggest complex interactions between ARV therapy during pregnancy and inflammatory responses in the mother and at the maternofetal interface. Further studies are needed to define these interactions in more details and their potential clinical consequences.

## Concluding Remarks

There is considerable evidence that HIV infection alters the transfer of maternal immune factors to HIV-exposed newborns and young infants. A negative impact on the transfer of maternal antibodies through the placenta has been observed in many settings for many antigen specificities. However, some studies reported preserved transfer of antibodies to pathogen or vaccine antigens; the reason for these discrepant results is unknown. A better understanding of the molecular regulation of IgG transfer through the placenta and how it is influenced by HIV infection is clearly needed. Further studies to determine the clinical consequences of the decreased transfer of maternal antibodies are also needed. The fact that the concentration of antibodies is preserved in breast milk of HIV-infected women supports a compensatory role of breast-feeding in the protection of HIV-exposed infants. On the other hand, immune activation in HIV-infected pregnant women is associated with production of inflammatory cytokines at the maternofetal interface and with inflammatory responses in the newborn. The clinical consequences of these immune alterations could include premature delivery, increased susceptibility to severe infections, and impaired neurological development. Immune activation may also play a role in the decreased transfer of maternal antibodies. The two factors may, therefore, interact and impact the health of HIV-exposed infants. Maternal immunization offers the potential to compensate for the decreased transfer of maternal antibodies. However, the impact of maternal HIV infection on vaccine responses, on the transfer of maternal antibodies through the placenta and breast milk, and on their waning in infants is likely to vary according the vaccine used and should be evaluated on a case-by-case basis. Finally, data suggest complex interactions between ARV prophylaxis during pregnancy and inflammatory responses in the mother and at the maternofetal interface. More studies are needed to clarify them and their possible clinical consequences. Despite about 20 years of research, the impact of maternal HIV infection on the health of HEU children remains poorly understood.

## Author Note

AM is a research Director of the Fund for Scientific Research, Belgium.

## Author Contributions

All of the authors met the following criteria: substantial contribution to the conception or design of the review, acquisition and review of the literature, drafting and revising of the final manuscript, and final approval of the version to be published.

## Conflict of Interest Statement

The authors declare that the research was conducted in the absence of any commercial or financial relationships that could be construed as a potential conflict of interest. The reviewer HJ and handling editor declared their shared affiliation, and the handling editor states that the process nevertheless met the standards of a fair and objective review.
